# Advances and prospects of multi-modal ophthalmic artificial intelligence based on deep learning: a review

**DOI:** 10.1186/s40662-024-00405-1

**Published:** 2024-10-01

**Authors:** Shaopan Wang, Xin He, Zhongquan Jian, Jie Li, Changsheng Xu, Yuguang Chen, Yuwen Liu, Han Chen, Caihong Huang, Jiaoyue Hu, Zuguo Liu

**Affiliations:** 1 Institute of Artificial Intelligence, Xiamen University, Xiamen, Fujian China; 2 School of Informatics, Xiamen University, Xiamen, Fujian China; 3 Xiamen University Affiliated Xiamen Eye Center, Fujian Provincial Key Laboratory of Ophthalmology and Visual Science, Fujian Engineering and Research Center of Eye Regenerative Medicine, Eye Institute of Xiamen University, School of Medicine, Xiamen University, Chengyi Building, 4Th Floor, 4221-122, South Xiang’an Rd, Xiamen, 361005 Fujian China; 4 Department of Ophthalmology, the First Affiliated Hospital of Xiamen University, Xiamen University, Xiamen, Fujian China; 5 National Engineering Research Center of Ophthalmology and Optometry, Eye Hospital, Wenzhou Medical University, Wenzhou, China; 6 Department of Ophthalmology, Xiang’an Hospital of Xiamen University, Xiamen, Fujian China

**Keywords:** Multi-modal ophthalmic research, Deep learning, Artificial intelligence

## Abstract

**Background:**

In recent years, ophthalmology has emerged as a new frontier in medical artificial intelligence (AI) with multi-modal AI in ophthalmology garnering significant attention across interdisciplinary research. This integration of various types and data models holds paramount importance as it enables the provision of detailed and precise information for diagnosing eye and vision diseases. By leveraging multi-modal ophthalmology AI techniques, clinicians can enhance the accuracy and efficiency of diagnoses, and thus reduce the risks associated with misdiagnosis and oversight while also enabling more precise management of eye and vision health. However, the widespread adoption of multi-modal ophthalmology poses significant challenges.

**Main text:**

In this review, we first summarize comprehensively the concept of modalities in the field of ophthalmology, the forms of fusion between modalities, and the progress of multi-modal ophthalmic AI technology. Finally, we discuss the challenges of current multi-modal AI technology applications in ophthalmology and future feasible research directions.

**Conclusion:**

In the field of ophthalmic AI, evidence suggests that when utilizing multi-modal data, deep learning-based multi-modal AI technology exhibits excellent diagnostic efficacy in assisting the diagnosis of various ophthalmic diseases. Particularly, in the current era marked by the proliferation of large-scale models, multi-modal techniques represent the most promising and advantageous solution for addressing the diagnosis of various ophthalmic diseases from a comprehensive perspective. However, it must be acknowledged that there are still numerous challenges associated with the application of multi-modal techniques in ophthalmic AI before they can be effectively employed in the clinical setting.

**Supplementary Information:**

The online version contains supplementary material available at 10.1186/s40662-024-00405-1.

## Background

In this rapidly advancing technology landscape, artificial intelligence (AI) has emerged as a pivotal catalyst for societal progress. The ubiquitous adoption of AI technologies spans diverse industries with machine learning and deep learning (DL) standing out as the most dynamic and transformative branches within the field of AI. Machine learning relies on data-driven methodologies wherein autonomous task execution is achieved by extracting patterns and regularities from extensive datasets.

**Fig. 1:**
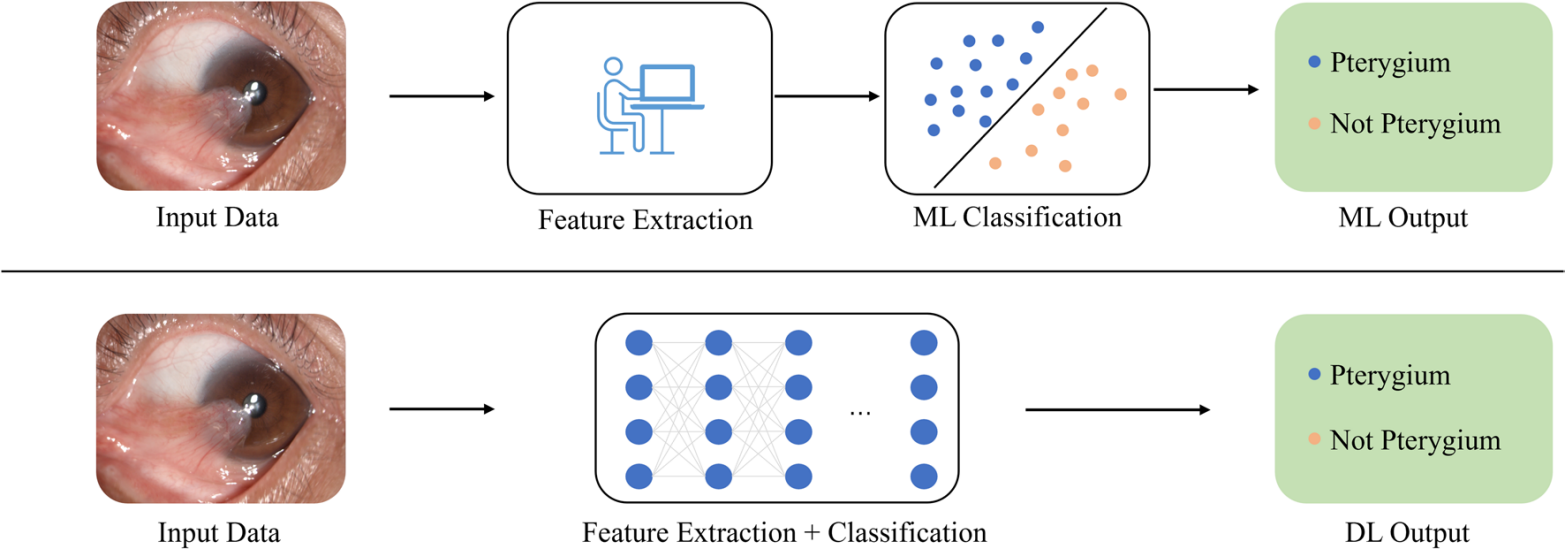
Paradigm comparison of machine learning and deep learning. Top row: general paradigm for machine learning. Bottom row: paradigm for deep learning. The example shown here is the classification of pterygium. ML, machine learning; DL, deep learning

DL [1], as a branch of machine learning, achieves efficient processing and abstract representation of complex data by constructing multi-layer neural network models that simulate the structure and functionality of the human brain. This approach enables end-to-end training on large-scale datasets (Fig. 1).

DL has found widespread application in the field of ophthalmology such as blink detection [2], eye movement tracking [3], diagnosis of ophthalmic diseases [4], and utilizing ocular images as systemic biomarkers to predict parameters of organs like the liver, kidneys, and blood [5]. Furthermore, retinal fundus image analysis has been employed to predict cardiovascular risk factors [6], forecast pathogenic genes in hereditary retinal diseases [7, 8], facilitate patient care and clinical decision-making through electronic medical record processing [9], advance digital education in ophthalmology [10], aid in the development and management of ophthalmic drugs [11], referral recommendation [12], and enable robotic surgical procedures in ophthalmology [13].

## Main text

### Concept of multimodality

Humans can interact with or perceive the world through various sensory organs, such as vision, hearing, touch, taste, and more. Information obtained through different pathways or forms is often referred to as different modalities. Generally, multi-modal machine learning refers to the construction of machine learning models capable of processing information from multiple modalities. Common modalities include vision, text, and speech [14].

**Fig. 2:**
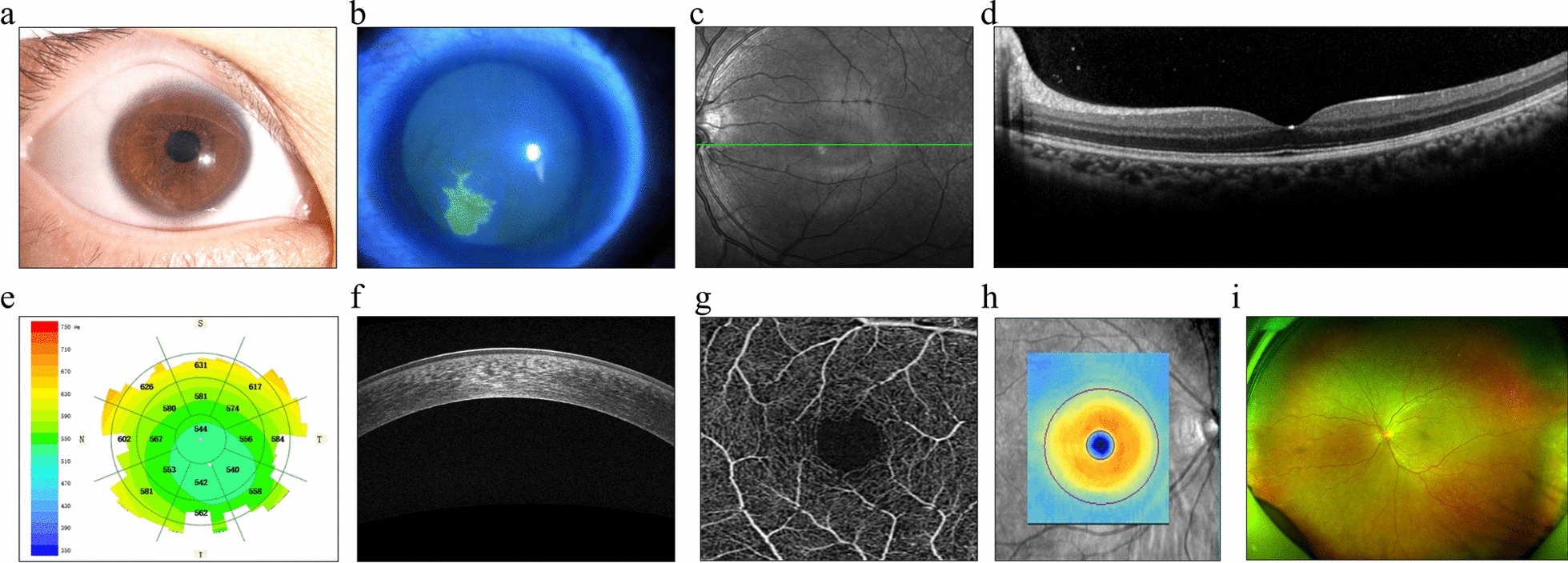
Presentation of typical multimodal ophthalmic imaging. The ophthalmic image-assisted examinations, arranged from left to right in the top row, include slit-lamp examination (**a**), corneal fluorescein sodium staining microscopy (**b**), and fundus optical coherence tomography (OCT) scan (**c** and **d**). In the bottom row, arranged from left to right, examinations consist of corneal epithelial thickness measurement (**e**), corneal OCT scan (**f**), fundus OCT angiography (OCTA) (**g**), ganglion cell examination (**h**), and wide-angle fundus color photography (**i**)

Compared to a single modality, multi-modal DL can provide the model with a greater variety of learnable data features. It enables the processing of different information extractions during the neural network learning phase, facilitating the effective fusion of multiple modalities. Figure 2 illustrates ophthalmic imaging from different modalities.

### Multi-modal fusion

#### Conventional fusion strategies

Early multi-modal fusion methods are categorized into feature-level, decision-level, and hybrid-level fusion. Feature-level fusion, or early fusion, combines modality features into a joint representation for decision-making [15, 16], while decision-level fusion, or late fusion, predicts results from unimodal features and then combines these results [17]. Hybrid-level fusion merges the benefits of both approaches for improved performance [18].

**Fig. 3:**
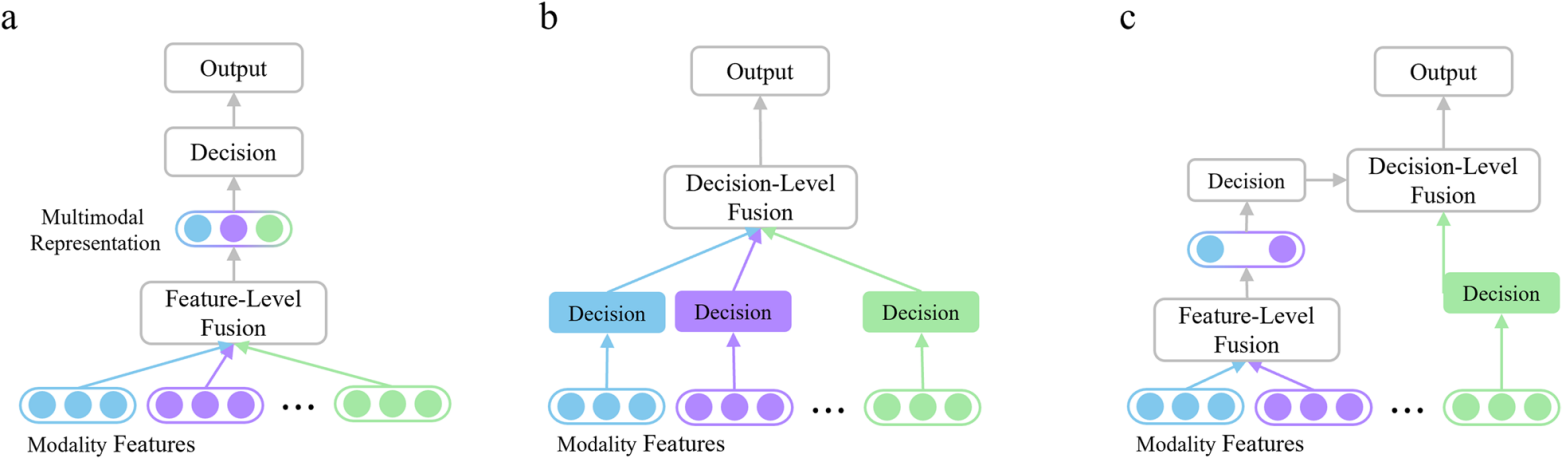
Different modality fusion strategies. **a** Feature-level fusion methods; **b** Decision-level fusion methods; **c** Hybrid-level fusion

Figure 3a shows that feature-level fusion methods extract and combine features from input modality signals to create an informative representation for decision-making. These methods integrate features from different modalities to generate a robust multi-modal representation, which has been extensively studied for its potential to handle noise and redundancy. Various algorithms, including machine learning, statistical techniques like principle component analysis (PCA) and independent component analysis (ICA), and DL models [19] have been proposed to enhance feature-level fusion performance.

Conversely, Fig. 3b illustrates decision-level fusion methods, which combine decisions or classification results from multiple input signals to improve accuracy and robustness. The key aspect here is the fusion rule, such as weighted averaging, majority voting, or Dempster-Shafer theory [20]. For example, in medical image analysis, multiple imaging modalities like magnetic resonance imaging (MRI), computed tomography (CT), and ultrasound provide classification decisions that are combined using a fusion rule to produce a final diagnosis [21].

Feature-level fusion focuses on obtaining valid fused features but risks overfitting and poor cross-view dynamics modeling, while decision-level fusion excels at modeling view-specific dynamics and adapting to varying modalities but fails to fully explore dynamic interactions and low-level modality interactions. Hybrid-level fusion combines multiple fusion levels to enhance quality, accuracy, and robustness, as shown in Fig. 3c. It offers flexibility in designing and optimizing algorithms by allowing the selection and combination of different techniques at each level [18]. However, it is more complex and computationally expensive than individual fusion levels due to the need to implement and integrate multiple techniques.

#### Modality interaction strategies

To alleviate the defects of conventional fusion strategies, a series of modality interaction strategies have been developed to enhance the integration of diverse data sources and improve overall performance [22]. By leveraging advanced techniques such as attention mechanisms, cross-modal learning, and co-training, these strategies can better capture the complementary information from each modality, resulting in more comprehensive and reliable results.

With the rise of the self-attention mechanism, cross-attention modality interaction [23, 24] has gained prominence, enabling more sophisticated and effective integration of information across different modalities. This approach allows models to dynamically attend to relevant features from multiple sources, improving the accuracy and robustness of multi-modal tasks by leveraging the strengths of each modality in a coordinated manner. In addition, convolution plays a potential role in multi-modal interaction, providing a means to capture local dependencies and spatial hierarchies within the data by viewing different modality signals as multiple channels and then enhancing the fusion process by efficiently integrating information across convolution kernels [25, 26].

Deep neural network methods excel in data fusion due to their ability to leverage vast datasets for learning. Modern neural architectures also facilitate seamless end-to-end training encompassing both multi-modal representation and fusion components. They typically outperform systems not based on neural networks and can learn intricate decision boundaries that prove difficult for other methods.

### Multi-modal AI in ophthalmology

#### Multi-modal AI and glaucoma

Glaucoma is one of the leading causes of irreversible blindness worldwide, characterized by structural damage and functional loss [27]. There have been many studies on single-modality glaucoma, such as optic cup and disc segmentation [28–30], glaucoma diagnosis and progression risk assessment based on fundus photography [31], and smartphone-based glaucoma detection systems [32]. Mehta et al. trained a multi-modal network using optical coherence tomography (OCT) and fundus photography clinical data from 1193 eyes of 863 healthy subjects and 1283 eyes of 771 glaucoma patients in the UK Biobank database [33]. The multi-modal model, which combined images, demographics, and clinical features, achieved high performance with an area under the curve (AUC) of 0.97 [34]. However, there is evidence suggesting that the database exhibits a slight bias towards healthy volunteers, which may lead to a bias in the trained model when applied to the general population [35]. Xiong et al. used visual field reports and peripapillary circular OCT scans to construct a fusion model (FusionNet) for detecting glaucomatous optic neuropathy. Compared with glaucoma experts, the fusion model achieved an AUC of 0.95 [36]. Since the data for these studies were derived from hospitalized patients, further validation of the algorithm's efficacy with the general population is still required. Huang et al. constructed the Glaucoma Real-world Progression Evaluation (GRAPE) dataset, which contains 1115 follow-up records of 263 eyes, including visual fields, fundus images, intraocular pressure, OCT measurements, and other multi-modal information. The team used a ResNet-50 model to demonstrate the feasibility of predicting visual field loss and progression, which can be used to evaluate glaucoma progression [37]. The Zhongshan Ophthalmic Center (ZOC) released the Glaucoma Automated Multi-Modality Platform for Diagnosis and Progression Assessment (GAMMA), a multi-modal dataset consisting of 2D fundus images and 3D OCT images of 300 patients for glaucoma grading. The dataset includes three tasks: glaucoma grading using multi-modal data, macular fovea detection using fundus images, and optic cup and disc segmentation [38]. This is a relatively comprehensive public dataset for the assessment of glaucoma, and it has served as the foundation for multitude of research endeavors. Wu et al. used GAMMA to construct a fusion model that can detect normal, early glaucoma, and advanced glaucoma [39]. Zhou et al. proposed a multi-modal universal architecture (MM-RAF) based on transformer, which uses self-attention mechanism and consists of three modules: bilateral contrastive alignment (BCA) aligns two modalities to the same semantic space to bridge semantic gaps; multiple instance learning representation (MILR) integrates multiple OCT scans into one semantic structure and reduces the OCT branch; and hierarchical attention fusion uses spatial information to enhance cross-modal interaction. Using these three modules, this architecture can effectively handle cross-modal information interaction with huge differences. They demonstrated that this design outperforms existing multi-modal methods in glaucoma recognition tasks, even on small clinical datasets [40, 41]. To address the problem of scarce multi-modal research data for glaucoma, Luo et al. proposed two solutions. First, the team developed a pseudo-supervised generalization-enhanced semi-supervised learning (SSL) model that optimized the pseudo-label prediction strategy for unlabeled samples to make the best use of unlabeled data and improve the model’s generalization ability. The results showed that the model outperformed the state-of-the-art (SOTA) of SSL comparison models. Second, the team established and publicly released the Harvard Glaucoma Detection and Progression (Harvard GDP) dataset of 1000 patients with multi-modal data [42], which is the first publicly available dataset for glaucoma progression prediction. It is believed that the release of this dataset can promote multi-modal research on glaucoma [43]. A summary of recent research in multi-modal approaches for glaucoma is presented in Table 1. The more precise information, including details on data processing, data augmentation, loss functions, learning rates, and other such specifics, is also summarized in Table S1.

**Table 1:** A summary of studies utilizing multimodal AI approaches in glaucoma

Year	Author	Task	Multimodal data types	Dataset scale	Dataset availability
2021	Mehta P et al. [34]	Glaucoma detection	Fundus images, OCT images	1,283 eyes of 771 glaucoma patients	Upon request
2022	Xiong J et al. [36]	Glaucomatous optic neuropathy	Visual field reports, peripapillary circular OCT scans	2,463 pairs of VF and OCT images from 1083 patients	Private dataset
2023	Huang X et al. [37]	Glaucoma management	Visual field, fundus images, intraocular pressure, OCT images	1,115 follow-up records of 263 eyes	Freely available in https://springernature.figshare.com/collections/GRAPE_A_multimodal_glaucoma_dataset_of_followup_visual_field_and_fundus_images_for_glaucoma_management/6406319/1
2021	Wu J et al. [38]	Glaucoma grading	Fundus images, OCT images	300 patients	Free available after registration in https://gamma.grandchallenge.org/
2023	Wu J et al. [39]	Glaucoma grading	Fundus images, OCT images	300 patients	Free available after registration in https://gamma.grand-challenge.org/
2023	Zhou Y et al. [40]	Glaucoma recognition	Fundus images, OCT images	1,200 images	Free available after registration in https://ichallenges.grand-challenge.org/iChallenge-PM/
2023	Luo Y et al. [43]	Glaucoma detection and progression forecasting	OCT images of glaucoma detection and progression	1,000 samples from 1,000 patients	Free download after approval

*OCT* = optical coherence tomography

#### Multi-modal AI and age-related macular degeneration

Age-related macular degeneration (AMD) is considered a primary cause of visual impairment in individuals aged 60 years and above. It can be classified into two types: dry and wet AMD [44]. Wang et al. collected fundus color photographs and OCT images of AMD patients, constructing a dual-stream convolutional neural network (DCNN) model for extracting OCT and fundus color photograph features. Subsequently, these features were concatenated and input into a classification layer for a three-class classification of normal fundus, dry AMD, and wet AMD [45]. Vaghefi et al. recruited 75 subjects divided into young healthy, elderly healthy, and moderate dry AMD patient groups. They collected fundus color photographs, OCT, and optical coherence tomography angiography (OCTA) imaging data from the participants. Using OCT and OCTA separately, they achieved diagnostic accuracies of 94% and 91%, respectively, and achieved a combined accuracy of 96% when utilizing multiple modalities [46]. Xu et al. collected fundus color photographs and OCT images from patients and employed a dual-stream deep convolutional neural network model based on ResNet-50 (DCNN) to identify AMD and polypoidal choroidal vasculopathy (PCV). They tested the model on 143 paired fundus and OCT images, achieving an accuracy of 87.4% [47]. However, the data imbalance between dry AMD and the remaining categories may affect the actual performance of the model. Chen and colleagues conducted a dual-center retrospective study in which they collected 2006 paired images of infrared reflectance (IR) and OCT. They designed a feature fusion method based on ResNet50 for vertical plane feature fusion (VPFF). The results demonstrated an accuracy of 0.9659 for the identification of dry AMD and 0.9930 for wet AMD on an external validation set, with an overall AUC of 0.9944. They posited that integrating the global information from IR and the local information from OCT significantly enhances the diagnostic accuracy of DL models [48]. Jin et al. conducted a retrospective cross-sectional multicenter study, including patients over 50 years old diagnosed with typical neovascular AMD. They collected 462 paired OCT and OCTA data points and developed a feature-level fusion model to detect choroidal neovascularization (CNV) in AMD patients [49]. Incorporating fundus fluorescein angiography (FFA) data into this study could further enhance the diagnosis of CNV. Patients diagnosed with exudative neovascular AMD typically undergo anti-vascular endothelial growth factor (anti-VEGF) drug therapy. To objectively assess the treatment response, Chorev and colleagues collected clinical characteristics and OCT scans from 1720 eyes of 1612 patients. They trained a multi-modal AI system that, compared to random selection and other standards, yielded superior results in predicting treatment response. This multi-modal AI-driven queue selection tool contributes to the more effective design of clinical trials for novel interventions and provides an objective theoretical basis for personalized care [50]. Song and collaborators developed a functional small animal retinal imaging system that includes polarization-sensitive OCT (PS-OCT), fluorescence scanning laser ophthalmoscopy (fSLO), and sensorless adaptive optics (SAO). This system facilitates the visualization of pathological features, and the newly developed system offers a more comprehensive information perspective for AMD detection from a multi-modal approach [51]. Moreover, there are several publicly available AMD multi-modal datasets, such as Age-Related Eye Disease Study (AREDS and AREDS II) [52], which provide additional opportunities for research in this field. However, access to these datasets requires the submission of a comprehensive research proposal and subsequent approval. Table 2 shows a summary of recent research in multi-modal approaches for AMD. The details on data processing and training parameters are summarized in Table S2.

**Table 2:** Summary of studies utilizing multimodal AI approaches in age-related macular degeneration (AMD)

Year	Author	Task	Multimodal data types	Dataset scale	Dataset availability
2019	Wang W et al. [45]	AMD classification	Fundus images, OCT images	2270 images	Private dataset
2020	Vaghefi E et al. [46]	AMD classification	Fundus images, OCT images, OCTA images	75 subjects	Private dataset
2021	Xu Z et al. [47]	AMD and PCV classification	Fundus images, OCT images	1099 eyes	Private dataset
2022	Chen M et al. [48]	AMD classification	Infrared reflectance and OCT images	2006 paired images	Private dataset
2022	Jin K et al. [49]	Identification of choroidal neovascularization in AMD	OCT images, OCTA images	462 paired images	Private dataset
2023	Chorev M et al. [50]	Identification of suboptimal responders to anti-VEGF drugs from exudative neovascular AMD patient	Clinical characteristics and OCT images	1720 eyes of 1612 patients	Private dataset

*PCV* = polypoidal choroidal vasculopathy; *OCT* = optical coherence tomography; *OCTA* = optical coherence tomography angiography; *VEGF* = vascular endothelial growth factor

#### Multi-modal AI and diabetic retinopathy

Diabetic retinopathy (DR) is a common complication of diabetes, and if not promptly addressed, it may lead to visual impairment or even blindness [53]. Numerous research efforts have been conducted utilizing DL to detect features of DR [54]. Li et al. employed generative networks to synthesize fluorescein angiography (FA) modal data of the fundus vessels and combined it with fundus color photographs. They utilized a self-supervised neural network to learn modality feature invariance and task-specific features. The constructed network, testing on the Ichallenge-AMD dataset [55], Ichallenge-PM dataset [56], and EyePACS dataset [57], demonstrated the ability to acquire diagnostic information across different modalities, which shows effectiveness for fundus disease classification [58]. This study employs an unsupervised approach to automatically learn features for subsequent classification tasks. A notable limitation is the small sample size used for self-supervised learning. This raises concerns about whether the limited samples can sufficiently learn effective features for the practical clinical classification of AMD. He et al. proposed a modality-specific attention network (MSAN) based on fundus photography and OCT images for the classification of fundus retinal images. Two specific attention modules were employed to extract features from fundus images and OCT images, respectively. Subsequently, through a modality fusion module, complementary feature information was learned, resulting in accurate fundus image classification that surpassed the results of single-modal approaches [59]. When handling model outputs, it converts the multi-label classification task into multiple binary classification tasks. This conversion artificially disrupts the correlations between different retinal diseases, which is inconsistent with the clinical practice of doctors who consider the interconnections between various diseases. This issue should be given sufficient attention.

Li et al. utilized fundus color photographs and OCT images, selectively fusing these two modal features for multi-modal multi-instance learning (MM-MIL). This lightweight network was found to be suitable for learning from small-scale data. Testing on 1,206 multi-modal data from 1,193 eyes of 836 subjects confirmed the effectiveness of the proposed method in retinal disease recognition [60]. Hervella et al. introduced a novel self-supervised pre-training method that extracted both shared features among different modalities and unique features for each input modality. This comprehensive understanding of the input domain facilitated downstream tasks such as DR classification, and experimental results confirmed the efficacy of this pre-training approach [61] using a public multi-modal dataset [62]. EI Habib Daho et al. utilized ultra-widefield color fundus photography (UWF-CFP) images and OCTA images to employ a fusion model combining ResNet50 and 3D-ResNet50, incorporating a Squeeze-and-Excitation (SE) module [63] to enhance relevant features, achieving significant improvements in DR classification compared to single-modal approaches in a dataset of EviRed [64], and thus aid in early detection [65]. Li et al. developed and automatically detected proliferative DR model using multi-modal data obtained from 3D OCT, 3D OCTA, and 2D fundus microscopy. They investigated the impact of early fusion, mid fusion, and hierarchical fusion on multi-modal performance, confirming that multi-modal models outperformed single-modal ones. Additionally, hierarchical fusion yielded better results compared to other fusion methods [66]. The main limitation of this study is the small sample size. These conclusions need to be validated on a larger clinical dataset. Bidwai et al. released a multi-modal database containing 76 patients with 111 OCTA images and 111 fundus color photographs. This database includes three categories: non-DR, mild DR, and moderate DR [67, 68]. The public availability of this dataset provides more opportunities for research on DR. A summary of recent research in multi-modal approaches for DR is depicted in Table 3, and more details on data preprocessing and model training can be found in Table S3.

**Table 3:** Summary of studies utilizing multimodal AI approaches in diabetic retinopathy

Year	Author	Task	Multimodal data types	Dataset scale	Dataset availability
2020	Li X et al. [58]	Fundus disease classification	Fundus images, synthesized FFA images	1200 images in [55]; 1200 images in [56]; 88,702 images in [57]	Free available after registration in https://ichallenges.grand-challenge.org/, https://www.kaggle.com/competitions/diabetic-retinopathy-detection/data
2021	He X et al. [59]	Fundus disease classification	Fundus images, OCT images	933 eyes of 498 patients	Private dataset
2021	Li X et al. [60]	Retinal disease Recognition	Fundus images, OCT images	1,193 eyes of 836 subjects	Private dataset
2022	Hervella Á et al. [61]	Diabetic retinopathy classification	Fluorescein angiography and color retinography	59 multimodal image pairs	Freely available from http://misp.mui.ac.ir/data/eye-images.html
2023	El Habib Daho M et al. [65]	Diabetic retinopathy classification	Ultra-widefield color fundus images and OCTA images	875 eyes from 444 patients	Upon request
2023	Li Y et al. [66]	Detection of proliferative diabetic retinopathy	OCT and OCTA images, fundus images	64 patients with diabetes	Private dataset
2024	Bidwai P et al. [67]	Diabetic classification	OCTA images and fundus images	222 images of 76 people	Upon request

*FFA* = fundus fluorescein angiography; *OCT* = optical coherence tomography; *OCTA* = optical coherence tomography angiography

### Potential challenges and future directions

**Fig. 4:**
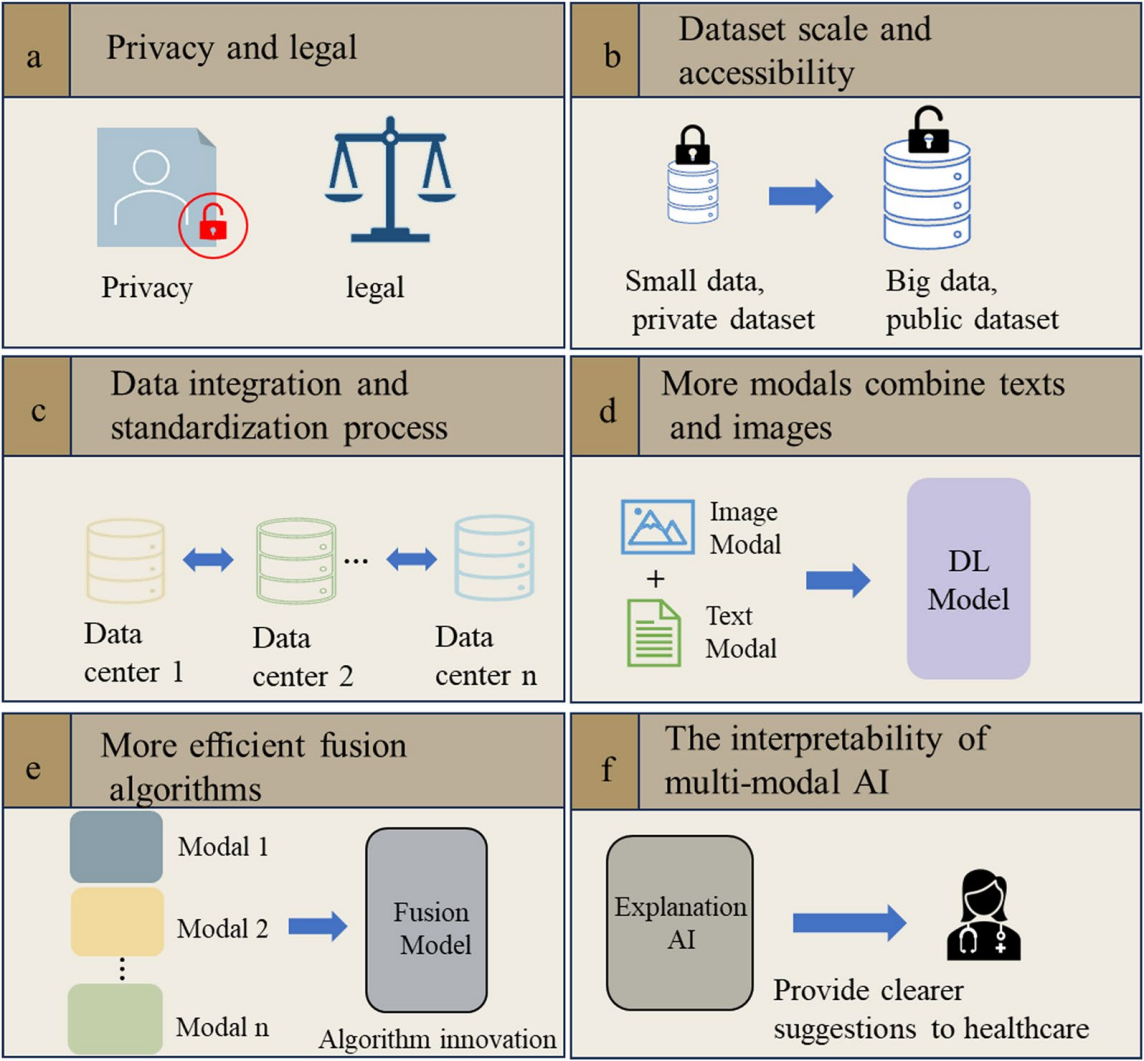
Potential challenges and future directions of multi-modal AI in ophthalmology. DL, deep learning; AI artificial intelligence.

Based on DL, multi-modal ophthalmic AI applications have made remarkable progress, especially in glaucoma and fundus diseases. However, there are still many challenges that need to be addressed (Fig. 4).

#### Privacy and legal

The application of DL-based AI technologies has demonstrated mature systems and products. However, in the context of medical applications, it is imperative to prioritize patient privacy rights [69]. Additionally, ethical, legal, and other pertinent issues should be thoroughly addressed concerning the involvement of AI products in healthcare scenarios. Furthermore, the iris structure of adult humans exhibits a high degree of uniqueness and stability, which remains unchanged unless subjected to trauma or surgical intervention. Consequently, automated acquisition and comparison of iris images through computer technology enable precise identification and authentication of individual identity information, a process referred to as iris recognition. Despite extensive research efforts dedicated to the development and optimization of iris recognition technology [70, 71] in recent years, it is undeniable that the potential leakage of biometric information may pose security risks [72].

In recent years, the emergence of federated learning [73] has presented significant prospects for addressing privacy concerns associated with medical data sources as well as potential legal and ethical issues. Serving as a pivotal technology within the realm of privacy-preserving computation, federated learning employs a mechanism wherein a central server trains a shared global model while keeping sensitive data stored locally within each participating institution, and thus ensure the preservation of privacy without disclosure.

#### Dataset scale and accessibility

It is well known that large-scale public datasets such as ImageNet [74], PASCAL VOC [75], COCO [76], and others play a significant role in boosting the performance of DL in various tasks related to natural image processing. It was observed that the majority of datasets utilized in the aforementioned studies for multi-modal ophthalmic AI were limited in size. Indeed, a considerable portion of the research work utilizes private datasets. The entire field of AI in ophthalmology lacks large-scale public datasets that can be freely provided to researchers.

To this end, further investigation is required to determine whether these smaller datasets adequately fulfill the requirements of real-world clinical applications. Additionally, more public datasets should be made open-source to promote the continuous development of the field.

#### Data integration and standardization process

Data quality is a critical factor for the success of DL models, impacting not only their accuracy but also directly influencing the reliability and efficiency of the models in addressing real-world problems [77]. The level of data quality determines whether the model can capture accurate features and whether these features can accurately reflect the complexity of the real world. If errors or biases exist in the dataset, the model may learn these inaccuracies during the training process, leading to distorted predictive results in practical applications. Indeed, factors such as economic level and the distribution of social opportunities can influence data bias, ultimately impacting the decision-making capabilities of algorithms [78]. Moreover, the quality of images in low-resource settings is another critical factor to consider, making it essential to obtain datasets from diverse communities to limit bias in data structure. To enhance the generalizability and fairness of models, it is crucial to include more samples from various economic backgrounds and resource levels in the training data.

Data preprocessing techniques, such as normalization [79] and data augmentation [80, 81], are essential for enhancing model performance. However, even with proper data preprocessing, if there is a mismatch in the distribution between test and training data, known as data distribution shift [82], the model's performance can be affected. In such cases, patterns learned during training may not be directly applicable to new and different distribution datasets. For example, a model trained well on images taken during the day may not perform optimally in low-light conditions due to inconsistent lighting distribution between the training and testing data.

To address these challenges, research groups must invest considerable effort in data collection, cleaning, labeling, and preprocessing stages. They need to ensure dataset diversity, handle missing values, correct errors, and assess the model's generalization ability through techniques like cross-validation [83]. Additionally, strategies such as transfer learning [84] can assist models in rapidly adapting to new domains, mitigating the impact of data distribution shift. Therefore, it is very important to further strengthen the cooperation mode of various units and establish a powerful data integration and standardization protocol.

#### More modals combine texts and images

Most of the research on multi-modal ophthalmology focuses on the interaction of information between different modal images, and less on the use of information from text. In fact, it should be noted that medical reports contain valuable supplementary diagnostic information and other contraindications, such as chief complaints, medical history, allergies, and so on. This portion of data has not been fully utilized.

Indeed, various studies have conducted in-depth investigations utilizing both text and images. Examples include the utilization of CT images and electronic health records for pulmonary metabolism detection [85], the generation of chest X-ray reports based on cross-modal multiscale feature fusion for pulmonary imaging [86], and the prediction of mortality rates in the ICU through the analysis of clinical records and temporal data [87]. Especially in the current era marked by the rise of large language models (LLM) [88, 89], the field of natural language processing (NLP) has witnessed enhanced capabilities in text comprehension and the extraction of textual features.

Recently, scholars have endeavored to employ LLM in the field of ophthalmology [90, 91]. Therefore, in the future, more consideration can be given to using valuable text features extracted from patients' rich medical history information combined with visual features of patient examinations to establish a more robust AI system.

#### More efficient fusion algorithms

Further research efforts are needed to explore more effectively and intelligently the selection of diverse features from multiple modalities, thereby enhancing the algorithmic level of clinical decision-making. This involves investigating advanced feature extraction techniques to ensure the comprehensive and contextually integrated incorporation of relevant information from various data sources. The pursuit of these directions will contribute to the development of more refined and efficient algorithms, ultimately bolstering the capabilities of the clinical decision-making process.

#### The interpretability of multi-modal AI

Over the years, AI-assisted systems have been commonly referred to as black-box systems [92], a characterization particularly pronounced in the context of DL. This implies that there is a lack of transparency regarding how DL systems make decisions and produce corresponding outputs. This opacity poses challenges to the widespread application of AI products in clinical settings [93].

However, the rapid development of interpretability in recent years, with emerging methods expanded the depth of this field. Attention mechanisms, such as attention rollout and attention flow [94], are prevalent interpretative tools that analyze attention distribution across multiple layers. These instruments aid in comprehending how information is transmitted across layers in DL models, particularly within transformer architectures. By employing these techniques, we can better visualize and quantify the decision-making process of models, thereby enhancing our understanding of model behavior. However, it is also noted by scholars that the direct application of attention weights in vision tasks has not yielded favorable outcomes [95]. Additionally, local interpretable model-agnostic explanation (LIME) is an interpretability technique [96] that generates synthetic data around an instance and learns a simpler, interpretable model through random perturbation to explain the predictions of a model on an individual sample. Shapley additive explanations (SHAP) have been proven to be an effective method [97] for constructing interpretable models, with a primary focus on computing the marginal contribution of features to model output, where Shapley values represent the average of marginal contributions across all possible combinations. SHAP offers a consistent and interpretable means to understand the importance of features. For some other convolutional neural network (CNN) models, gradient-based interpretability methods are also widely used. These methods include techniques operating on intermediate network layers [98] or modifications of the backpropagation rules [95]. While they are computationally efficient for most network architectures, gradient-based explanations have yielded mixed results in quantitative benchmark tests. Additionally, empirical evidence has shown that they are not sensitive to randomization of model parameters [99].

Enhancing the interpretability of AI decisions is essential for establishing trust among clinicians. This holds significant significance in the medical domain because the ability to comprehend and elucidate the decision-making process of AI is paramount for gaining the confidence of healthcare professionals in their clinical decision-making. When physicians possess a comprehensive understanding of the recommendations or decisions generated by AI systems, there is a higher likelihood of them embracing and implementing this information, ultimately elevating the standard and safety of medical decisions.

## Conclusions

DL currently serves as the cornerstone technology in AI within the field of ophthalmology, demonstrating significant advancements in research. Recent studies indicate that particularly in the auxiliary diagnosis of glaucoma and various fundus diseases, multi-modal learning exhibits notable advantages in performance outcomes compared to unimodal approaches. Multi-modal learning integrates information from diverse imaging modalities, supplying more comprehensive and multi-faceted data, thereby offering more accurate support for ophthalmic diagnostics. This approach exhibits immense potential in enhancing accuracy, early disease detection, and the formulation of personalized treatment plans. Consequently, the development of multi-modal DL techniques in ophthalmology holds vast prospects for application, providing robust tools to improve the efficiency and precision of ophthalmic healthcare. This trajectory not only anticipates driving innovation in the field of ophthalmology but also promises to deliver more personalized and advanced medical services to patients.

## Supplementary Information

Additional file 1.

Additional file 2.

Additional file 3.

## Data Availability

The article did not generate any data that can be provided.

## Abbreviations

DL: Deep learning
AI: Artificial intelligence
ML: Machine learning
AMD: Age-related macular degeneration
DR: Diabetic retinopathy
OCT: Optical coherence tomography
OCTA: Optical coherence tomography angiography
FFA: Fluorescein fundus angiography
LSTM: Long short-term memory
AUC: Area under the curve
NLP: Natural language processing
LLM: Large language model

## References

[1] LeCun Y, Bengio Y, Hinton G. Deep learning. Nature. 2015;521(7553):436–44. 26017442 10.1038/nature14539

[2] Zheng Q, Wang L, Wen H, Ren Y, Huang S, Bai F, et al. Impact of incomplete blinking analyzed using a deep learning model with the Keratograph 5M in dry eye disease. Transl Vis Sci Technol. 2022;11(3):38. 35357395 10.1167/tvst.11.3.38 PMC8976934

[3] Stember JN, Celik H, Krupinski E, Chang PD, Mutasa S, Wood BJ, et al. Eye tracking for deep learning segmentation using convolutional neural networks. J Digit Imaging. 2019;32(4):597–604. 31044392 10.1007/s10278-019-00220-4 PMC6646645

[4] Sengupta S, Singh A, Leopold HA, Gulati T, Lakshminarayanan V. Application of deep learning in fundus image processing for ophthalmic diagnosis--a review. arXiv preprint arXiv:181207101. 2018.

[5] Babenko B, Mitani A, Traynis I, Kitade N, Singh P, Maa AY, et al. Detection of signs of disease in external photographs of the eyes via deep learning. Nat Biomed Eng. 2022;6(12):1370–83. 35352000 10.1038/s41551-022-00867-5 PMC8963675

[6] Poplin R, Varadarajan AV, Blumer K, Liu Y, McConnell MV, Corrado GS, et al. Prediction of cardiovascular risk factors from retinal fundus photographs via deep learning. Nat Biomed Eng. 2018;2(3):158–64. 31015713 10.1038/s41551-018-0195-0

[7] Fujinami-Yokokawa Y, Pontikos N, Yang L, Tsunoda K, Yoshitake K, Iwata T, et al. Prediction of causative genes in inherited retinal disorders from spectral-domain optical coherence tomography utilizing deep learning techniques. J Ophthalmol. 2019;2019:1691064. 31093368 10.1155/2019/1691064 PMC6481010

[8] Fujinami-Yokokawa Y, Ninomiya H, Liu X, Yang L, Pontikos N, Yoshitake K, et al. Prediction of causative genes in inherited retinal disorder from fundus photography and autofluorescence imaging using deep learning techniques. Br J Ophthalmol. 2021;105(9):1272–9. 33879469 10.1136/bjophthalmol-2020-318544 PMC8380883

[9] Lin WC, Chen JS, Chiang MF, Hribar MR. Applications of artificial intelligence to electronic health record data in ophthalmology. Transl Vis Sci Technol. 2020;9(2):13. 32704419 10.1167/tvst.9.2.13 PMC7347028

[10] Al-Khaled T, Acaba-Berrocal L, Cole E, Ting DSW, Chiang MF, Chan RVP. Digital education in ophthalmology. Asia Pac J Ophthalmol (Phila). 2022;11(3):267–72. 34966034 10.1097/APO.0000000000000484 PMC9240107

[11] Feng D, Chen X, Zhou Z, Liu H, Wang Y, Bai L, et al. A preliminary study of predicting effectiveness of anti-VEGF injection using OCT images based on deep learning. In: 2020 42nd annual international conference of the IEEE engineering in medicine & biology society (EMBC); 2020: IEEE. 10.1109/EMBC44109.2020.9176743 33019208

[12] De Fauw J, Ledsam JR, Romera-Paredes B, Nikolov S, Tomasev N, Blackwell S, et al. Clinically applicable deep learning for diagnosis and referral in retinal disease. Nat Med. 2018;24(9):1342–50. 30104768 10.1038/s41591-018-0107-6

[13] Keller B, Draelos M, Zhou K, Qian R, Kuo A, Konidaris G, et al. Optical coherence tomography-guided robotic ophthalmic microsurgery via reinforcement learning from demonstration. IEEE Trans Robot. 2020;36(4):1207–18. 36168513 10.1109/TRO.2020.2980158 PMC9511825

[14] Baltrušaitis T, Ahuja C, Morency LP. Multimodal machine learning: a survey and taxonomy. IEEE Trans Pattern Anal Mach Intell. 2019;41(2):423–43. 29994351 10.1109/TPAMI.2018.2798607

[15] Tsai YHH, Bai S, Liang PP, Kolter JZ, Morency LP, Salakhutdinov R. Multimodal transformer for unaligned multimodal language sequences. In: Proceedings of the Conference Association for Computational Linguistics Meeting; 2019: NIH Public Access. 10.18653/v1/p19-1656 PMC7195022 32362720

[16] Zadeh A, Liang PP, Poria S, Vij P, Cambria E, Morency LP. Multi-attention recurrent network for human communication comprehension. In: Proceedings of the AAAI Conference on Artificial Intelligence; 2018. PMC7136010 32257595

[17] Kampman O, Barezi EJ, Bertero D, Fung P. Investigating audio, visual, and text fusion methods for end-to-end automatic personality prediction. arXiv preprint arXiv:180500705. 2018.

[18] Yu S, He M, Nie R, Wang C, Wang X. An unsupervised hybrid model based on CNN and ViT for multimodal medical image fusion. In: 2021 2nd International Conference on Electronics, Communications and Information Technology (CECIT); 2021: IEEE.

[19] Ehatisham-Ul-Haq M, Javed A, Azam MA, Malik HM, Irtaza A, Lee IH, et al. Robust human activity recognition using multimodal feature-level fusion. IEEE Access. 2019;7:60736–51.

[20] Kaur H, Koundal D, Kadyan V. Image fusion techniques: a survey. Arch Comput Methods Eng. 2021;28(7):4425–47. 33519179 10.1007/s11831-021-09540-7 PMC7829034

[21] Dogra A, Goyal B, Agrawal S. Medical image fusion: a brief introduction. Biomed Pharmacol J. 2018;11(3):1209.

[22] Hallitschke VJ, Schlumberger T, Kataliakos P, Marinov Z, Kim M, Heiliger L, et al. Multimodal interactive lung lesion segmentation: a framework for annotating PET/CT images based on physiological and anatomical cues. In: 2023 IEEE 20th International Symposium on Biomedical Imaging (ISBI); 2023: IEEE.

[23] Chowdhury MH, Chowdhury ME, Alqahtani A. MMG-net: multi-modal approach to estimate blood glucose using multi-stream and cross modality attention. Biomed Signal Process Control. 2024;92:105975.

[24] Zhang J, He X, Liu Y, Cai Q, Chen H, Qing L. Multi-modal cross-attention network for Alzheimer’s disease diagnosis with multi-modality data. Comput Biol Med. 2023;162:107050. 37269680 10.1016/j.compbiomed.2023.107050

[25] Bardak B, Tan M. Improving clinical outcome predictions using convolution over medical entities with multimodal learning. Artif Intell Med. 2021;117:102112. 34127241 10.1016/j.artmed.2021.102112

[26] Georgescu MI, Ionescu RT, Miron AI, Savencu O, Ristea NC, Verga N, et al. Multimodal multi-head convolutional attention with various kernel sizes for medical image super-resolution. In: Proceedings of the IEEE/CVF Winter Conference on Applications of Computer Vision; 2023.

[27] Weinreb RN, Aung T, Medeiros FA. The pathophysiology and treatment of glaucoma: a review. JAMA. 2014;311(18):1901–11. 24825645 10.1001/jama.2014.3192 PMC4523637

[28] Lim G, Cheng Y, Hsu W, Lee ML. Integrated optic disc and cup segmentation with deep learning. In: 2015 IEEE 27th International Conference on Tools with Artificial Intelligence (ICTAI); 2015: IEEE.

[29] Kim J, Tran L, Chew EY, Antani S. Optic disc and cup segmentation for glaucoma characterization using deep learning. In: 2019 IEEE 32nd International Symposium on Computer-Based Medical Systems (CBMS); 2019: IEEE.

[30] Bian X, Luo X, Wang C, Liu W, Lin X. Optic disc and optic cup segmentation based on anatomy guided cascade network. Comput Methods Programs Biomed. 2020;197:105717. 32957060 10.1016/j.cmpb.2020.105717

[31] Li F, Su Y, Lin F, Li Z, Song Y, Nie S, et al. A deep-learning system predicts glaucoma incidence and progression using retinal photographs. J Clin Invest. 2022;132(11):e157968. 35642636 10.1172/JCI157968 PMC9151694

[32] Li F, Song D, Chen H, Xiong J, Li X, Zhong H, et al. Development and clinical deployment of a smartphone-based visual field deep learning system for glaucoma detection. NPJ Digit Med. 2020;3:123. 33043147 10.1038/s41746-020-00329-9 PMC7508974

[33] UK Biobank. https://www.sciencedirect.com/topics/medicine-and-dentistry/uk-biobank. Accessed 16 May 2024.

[34] Mehta P, Petersen CA, Wen JC, Banitt MR, Chen PP, Bojikian KD, et al. Automated detection of glaucoma with interpretable machine learning using clinical data and multimodal retinal images. Am J Ophthalmol. 2021;231:154–69. 33945818 10.1016/j.ajo.2021.04.021 PMC8560651

[35] Fry A, Littlejohns TJ, Sudlow C, Doherty N, Adamska L, Sprosen T, et al. Comparison of sociodemographic and health-related characteristics of UK Biobank participants with those of the general population. Am J Epidemiol. 2017;186(9):1026–34. 28641372 10.1093/aje/kwx246 PMC5860371

[36] Xiong J, Li F, Song D, Tang G, He J, Gao K, et al. Multimodal machine learning using visual fields and peripapillary circular OCT scans in detection of glaucomatous optic neuropathy. Ophthalmology. 2022;129(2):171–80. 34339778 10.1016/j.ophtha.2021.07.032

[37] Huang X, Kong X, Shen Z, Ouyang J, Li Y, Jin K, et al. GRAPE: a multi-modal dataset of longitudinal follow-up visual field and fundus images for glaucoma management. Sci Data. 2023;10(1):520. 37543686 10.1038/s41597-023-02424-4 PMC10404253

[38] Wu J, Fang H, Li F, Fu H, Lin F, Li J, et al. GAMMA: glaucoma grading from multi-modality images. Med Image Anal. 2023;90:102938. 37806020 10.1016/j.media.2023.102938

[39] Wu J, Fang H, Li F, Fu H, Lin F, Li J, et al. GAMMA challenge: glaucoma grading from multi-modality images. Med Image Anal. 2023;90:102938. 37806020 10.1016/j.media.2023.102938

[40] Zhou Y, Yang G, Zhou Y, Ding D, Zhao J. Representation, alignment, fusion: a generic transformer-based framework for multi-modal glaucoma recognition. In: Greenspan H, Madabhushi A, Mousavi P, Salcudean S, Duncan J, Syeda-Mahmood T, Taylor R, editors. International Conference on Medical Image Computing and Computer-Assisted Intervention. Cham: Springer; 2023. p. 704–13.

[41] iChallenge-GON. https://ichallenges.grand-challenge.org/iChallenge-GON. Accessed 16 May 2024.

[42] Harvard glaucoma detection and progression with 1000 samples (Harvard-GDP1000). https://ophai.hms.harvard.edu/datasets. Accessed 16 May 2024.

[43] Luo Y, Shi M, Tian Y, Elze T, Wang M. Harvard glaucoma detection and progression: a multimodal multitask dataset and generalization-reinforced semi-supervised learning. In; Proceedings of the IEEE/CVF International Conference on Computer Vision; 2023.

[44] Hernández-Zimbrón LF, Zamora-Alvarado R, Ochoa-De la Paz L, Velez-Montoya R, Zenteno E, Gulias-Cañizo R, et al. Age-related macular degeneration: new paradigms for treatment and management of AMD. Oxid Med Cell Longev. 2018;2018:8374647. 29484106 10.1155/2018/8374647 PMC5816845

[45] Wang W, Xu Z, Yu W, Zhao J, Yang J, He F, et al. Two-stream CNN with loose pair training for multi-modal AMD categorization. In: Medical Image Computing and Computer Assisted Intervention–MICCAI 2019: 22nd International Conference, Shenzhen, China, October 13–17, 2019, Proceedings, Part I 22; 2019: Springer.

[46] Vaghefi E, Hill S, Kersten HM, Squirrell D. Multimodal retinal image analysis via deep learning for the diagnosis of intermediate dry age-related macular degeneration: a feasibility study. J Ophthalmol. 2020;2020:7493419. 32411434 10.1155/2020/7493419 PMC7201607

[47] Xu Z, Wang W, Yang J, Zhao J, Ding D, He F, et al. Automated diagnoses of age-related macular degeneration and polypoidal choroidal vasculopathy using bi-modal deep convolutional neural networks. Br J Ophthalmol. 2021;105(4):561–6. 32499330 10.1136/bjophthalmol-2020-315817

[48] Chen M, Jin K, Yan Y, Liu X, Huang X, Gao Z, et al. Automated diagnosis of age-related macular degeneration using multi-modal vertical plane feature fusion via deep learning. Med Phys. 2022;49(4):2324–33. 35172022 10.1002/mp.15541

[49] Jin K, Yan Y, Chen M, Wang J, Pan X, Liu X, et al. Multimodal deep learning with feature level fusion for identification of choroidal neovascularization activity in age-related macular degeneration. Acta Ophthalmol. 2022;100(2):e512–20. 34159761 10.1111/aos.14928

[50] Chorev M, Haderlein J, Chandra S, Menon G, Burton BJL, Pearce I, et al. A multi-modal AI-driven cohort selection tool to predict suboptimal non-responders to aflibercept loading-phase for neovascular age-related macular degeneration: PRECISE Study Report 1. J Clin Med. 2023;12(8):3013. 37109349 10.3390/jcm12083013 PMC10142969

[51] Song J, Miao Y, Matsubara JA, Sarunic MV, Ju MJ. Multi-modal functional sensorless adaptive optics for small animal retinal imaging. In: European Conference on Biomedical Optics; 2023: Optica Publishing Group.

[52] Age-Related Eye Disease Study (AREDS). https://www.nei.nih.gov/research/clinical-trials/age-related-eye-disease-studies-aredsareds2. Accessed 17 May 2024.

[53] Mohamed Q, Gillies MC, Wong TY. Management of diabetic retinopathy: a systematic review. JAMA. 2007;298(8):902–16. 17712074 10.1001/jama.298.8.902

[54] Alyoubi WL, Shalash WM, Abulkhair MF. Diabetic retinopathy detection through deep learning techniques: a review. Inform Med Unlocked. 2020;20:100377.

[55] iChallenge-AMD dataset. https://ichallenges.grand-challenge.org/iChallenge-AMD. Accessed 16 May 2024.

[56] IChallenge-PM dataset. https://ichallenges.grand-challenge.org/iChallenge-PM/. Accessed 16 May 2024.

[57] EyePACS dataset. https://www.kaggle.com/c/diabetic-retinopathy-detection/data. Accessed 16 May 2024.

[58] Li X, Jia M, Islam MT, Yu L, Xing L. Self-supervised feature learning via exploiting multi-modal data for retinal disease diagnosis. IEEE Trans Med Imaging. 2020;39(12):4023–33. 32746140 10.1109/TMI.2020.3008871

[59] He X, Deng Y, Fang L, Peng Q. Multi-modal retinal image classification with modality-specific attention network. IEEE Trans Med Imaging. 2021;40(6):1591–602. 33625978 10.1109/TMI.2021.3059956

[60] Li X, Zhou Y, Wang J, Lin H, Zhao J, Ding D, et al. Multi-modal multi-instance learning for retinal disease recognition. In: Proceedings of the 29th ACM International Conference on Multimedia; 2021.

[61] Hervella ÁS, Rouco J, Novo J, Ortega M. Multimodal image encoding pre-training for diabetic retinopathy grading. Comput Biol Med. 2022;143:105302. 35219187 10.1016/j.compbiomed.2022.105302

[62] Hajeb Mohammad Alipour S, Rabbani H, Akhlaghi MR. Diabetic retinopathy grading by digital curvelet transform. Comput Math Methods Med. 2012;2012:761901. 23056148 10.1155/2012/761901 PMC3465990

[63] Hu J, Shen L, Sun G. Squeeze-and-excitation networks. In: Proceedings of the IEEE Conference on Computer Vision and Pattern Recognition; 2018.

[64] EviRed. https://evired.org/. Accessed 16 May 2024.

[65] El Habib DM, Li Y, Zeghlache R, Atse YC, Le Boité H, Bonnin S, et al. Improved automatic diabetic retinopathy severity classification using deep multimodal fusion of UWF-CFP and OCTA images international workshop on ophthalmic medical image analysis. Cham: Springer; 2023.

[66] Li Y, Hajj HA, Conze PH, Daho ME, Bonnin S, Ren H, et al. Multimodal information fusion for the diagnosis of diabetic retinopathy. arXiv preprint arXiv:230400003. 2023.

[67] Bidwai P, Gite S, Gupta A, Pahuja K, Kotecha K. Multimodal dataset using OCTA and fundus images for the study of diabetic retinopathy. Data Brief. 2024;52:110033. 38299103 10.1016/j.dib.2024.110033 PMC10828556

[68] Multimodal OCTA and fundus image dataset for detection of diabetic retinopathy. https://zenodo.org/records/8375220. Accessed 16 May 2024.

[69] Price WN 2nd, Cohen IG. Privacy in the age of medical big data. Nat Med. 2019;25(1):37–43. 30617331 10.1038/s41591-018-0272-7 PMC6376961

[70] Ding Y, Tian L, Han B, Wang H, Wang Y, Zheng JX. Achieving privacy-preserving iris identification via el gamal. Comput Mater Contin. 2019;61(2):727–38.

[71] Nguyen K, Proença H, Alonso-Fernandez F. Deep learning for iris recognition: a survey. ACM Comput Surv. 2024;56(9):1–35.

[72] Yang W, Wang S, Hu J, Ibrahim A, Zheng G, Macedo MJ, et al. A cancelable iris-and steganography-based user authentication system for the internet of things. Sensors (Basel). 2019;19(13):2985. 31284592 10.3390/s19132985 PMC6651016

[73] Xu J, Glicksberg BS, Su C, Walker P, Bian J, Wang F. Federated learning for healthcare informatics. J Healthc Inform Res. 2021;5(1):1–19. 33204939 10.1007/s41666-020-00082-4 PMC7659898

[74] Deng J, Dong W, Socher R, Li LJ, Li K, Fei-Fei L. Imagenet: a large-scale hierarchical image database. In: 2009 IEEE conference on computer vision and pattern recognition; 2009: IEEE.

[75] Visual Object Classes Challenge 2012. http://host.robots.ox.ac.uk/pascal/VOC/voc2012/. Accessed 15 May 2024.

[76] COCO Dataset. https://cocodataset.org/. Accessed 15 May 2024.

[77] Whang SE, Roh Y, Song H, Lee JG. Data collection and quality challenges in deep learning: a data-centric ai perspective. VLDB J. 2023;32(4):791–813.

[78] Cowgill B, Tucker CE. Economics, fairness and algorithmic bias. Pre J Econ Perspec. 2019. 10.2139/ssrn.3361280.

[79] Sun J, Cao X, Liang H, Huang W, Chen Z, Li Z. New interpretations of normalization methods in deep learning. In: Proceedings of the AAAI Conference on Artificial Intelligence; 2020.

[80] Mikołajczyk A, Grochowski M. Data augmentation for improving deep learning in image classification problem. In: 2018 international interdisciplinary PhD workshop (IIPhDW); 2018: IEEE.

[81] Shorten C, Khoshgoftaar TM. A survey on image data augmentation for deep learning. J Big Data. 2019;6(1):1–48. 10.1186/s40537-021-00492-0 PMC8287113 34306963

[82] Fang T, Lu N, Niu G, Sugiyama M. Rethinking importance weighting for deep learning under distribution shift. Adv Neural Inf Process Syst. 2020;33:11996–2007.

[83] Berrar D. Cross-Validation. 2019.

[84] Weiss K, Khoshgoftaar TM, Wang D. A survey of transfer learning. J Big Data. 2016;3(1):1–40.

[85] Huang SC, Pareek A, Zamanian R, Banerjee I, Lungren MP. Multimodal fusion with deep neural networks for leveraging CT imaging and electronic health record: a case-study in pulmonary embolism detection. Sci Rep. 2020;10(1):22147. 33335111 10.1038/s41598-020-78888-w PMC7746687

[86] Pan Y, Liu LJ, Yang XB, Peng W, Huang QS. Chest radiology report generation based on cross-modal multi-scale feature fusion. J Radia Res Appl Sci. 2024;17(1):100823.

[87] Niu K, Zhang K, Peng X, Pan Y, Xiao N. Deep multi-modal intermediate fusion of clinical record and time series data in mortality prediction. Front Mol Biosci. 2023;10:1136071. 36968273 10.3389/fmolb.2023.1136071 PMC10030980

[88] Chang Y, Wang X, Wang J, Wu Y, Yang L, Zhu K, et al. A survey on evaluation of large language models. ACM Trans Intell Syst Technol. 2024;15(3):1–45.

[89] Thirunavukarasu AJ, Ting DSJ, Elangovan K, Gutierrez L, Tan TF, Ting DSW. Large language models in medicine. Nat Med. 2023;29(8):1930–40. 37460753 10.1038/s41591-023-02448-8

[90] Bernstein IA, Zhang YV, Govil D, Majid I, Chang RT, Sun Y, et al. Comparison of ophthalmologist and large language model chatbot responses to online patient eye care questions. JAMA Netw Open. 2023;6(8):e2330320. 37606922 10.1001/jamanetworkopen.2023.30320 PMC10445188

[91] Betzler BK, Chen H, Cheng CY, Lee CS, Ning G, Song SJ, et al. Large language models and their impact in ophthalmology. Lancet Digit Health. 2023;5(12):e917–24. 38000875 10.1016/S2589-7500(23)00201-7 PMC11003328

[92] Ferretti A, Schneider M, Blasimme A. Machine learning in medicine: opening the new data protection black box. Eur Data Prot L Rev. 2018;4:320.

[93] Poon AI, Sung JJ. Opening the black box of AI-Medicine. J Gastroenterol Hepatol. 2021;36(3):581–4. 33709609 10.1111/jgh.15384

[94] Abnar S, Zuidema W. Quantifying attention flow in transformers. arXiv preprint arXiv:200500928. 2020.

[95] Chefer H, Gur S, Wolf L. Transformer interpretability beyond attention visualization. In: Proceedings of the IEEE/CVF conference on computer vision and pattern recognition; 2021.

[96] Zafar MR, Khan N. Deterministic local interpretable model-agnostic explanations for stable explainability. Mach Learn Knowl Extr. 2021;3(3):525–41.

[97] Nohara Y, Matsumoto K, Soejima H, Nakashima N. Explanation of machine learning models using shapley additive explanation and application for real data in hospital. Comput Methods Programs Biomed. 2022;214:106584. 34942412 10.1016/j.cmpb.2021.106584

[98] Selvaraju RR, Cogswell M, Das A, Vedantam R, Parikh D, Batra D. Grad-CAM: visual explanations from deep networks via gradient-based localization. In: Proceedings of the IEEE international conference on computer vision; 2017.

[99] Adebayo J, Gilmer J, Muelly M, Goodfellow I, Hardt M, Kim B. Sanity checks for saliency maps. In: Advances in Neural Information Processing Systems. 2018. p. 9505–15.

