# Supplementary material for: Advances and prospects of multi-modal ophthalmic artificial intelligence based on deep learning: a review
Source: Eye Vis (Lond). 2024 Oct 1;11:38. doi: 10.1186/s40662-024-00405-1 (PMC11443922; doi:10.1186/s40662-024-00405-1)
Supplement: Supplementary file 1 — Additional file 1. [file 40662_2024_405_MOESM1_ESM.docx]

**Table S1. A detail description of research in multimodal approaches for glaucoma.**

| Authors | Data  preprocessing | Data augmentation | Model descriptions | | | | | | |
| --- | --- | --- | --- | --- | --- | --- | --- | --- | --- |
|  |  |  | **Loss function** | **Learning**  **rate** | **Batch size** | **Epoch** | **Optimizer** | **Model process** | **Public code link** |
| Mehta P  et al. [34] | - Each right eye image was flipped left to right - Then, each scan was cropped to a ratio of 1:1 and down sampled to 224×224 pixels | Affine, elastic and intensity transformation | Cross-entropy | Initial learning rate is 1e-3/1e-5 | 80/400 | 60/20 | Adam | Gradient-boosted decision tree to build the final model | None |
| Xiong J et al. [36] | - OCT images were resized to 256×768 pixels - VF PDF reports were divided into 10×10 grids | None | Cross-entropy | Initial learning rate is 1e-3 | 32 | 120 | Adam | FusionNet consists of OCTNet and VFNet with an attention module to obtain aggerated representation | <https://github.com/dpsong/multimodalGlaucoma> |
| Huang X et al. [37] | - ROI was clipped from original CFP photographs - Annotations for the optic disc and optic cup was labeled by an ophthalmologist | None | Cross-entropy | Annealed cosine to update the learning rate from 1e-4 to 1e-5 | 4 | 264 | Adam | Resnet-50 model to show the technical validation of dataset | None |
| Junde Wu  et al. [38] | - Each fundus image was annotated manually by four clinical ophthalmologists | CenterCrop, RandomHorizontalFlip, RandomVertivalFlip | Cross-entropy | Initial learning rate is 1e-4 | 4 | 1000 tiers | Adam | The features from OCT network branch and fundus network branch are concatenated for the final classification | <https://aistudio.baidu.com/projectdetail/1948228> |
| Zhou Y et al. [40] | - A clinician relabels the test set as GON/normal by considering CFP and OCT thickness map | None | Cross-entropy, bilateral contrastive (BAC) loss | Initial learning rate is 1e-3 | 32 | 100 | SGD | A pure self-attention multi-modal framework consisting of three modules, BCA fills the semantic gap between CFP and OCT, MILR and HAF complete semantic aggregation and relationship probing | <https://github.com/YouZhouRUC/MM-RAF> |
| Luo Y et al. [43] | - Data quality control was applied to exclude unreliable data | MixMatch, ReMixMatch, FixMatch, DASO, random crop, resize | Cross-entropy | Learning rate is 4e-5 and weight decay of 0 | Vary because of various methods | 10 | AdamW | A newly developed model called pseudo supervisor was used to optimally utilize unlabeled data | <https://github.com/Harvard-Ophthalmology-AI-Lab/Harvard-GDP> |

OCT = optical coherence tomography; VF = visual fields; ROI = region of interest; CFP = color fundus photography; BCA = Bilateral Contrastive Alignment; GON = Glaucomatous optic neuropathy
