# Supplementary material for: Advances and prospects of multi-modal ophthalmic artificial intelligence based on deep learning: a review
Source: Eye Vis (Lond). 2024 Oct 1;11:38. doi: 10.1186/s40662-024-00405-1 (PMC11443922; doi:10.1186/s40662-024-00405-1)
Supplement: Supplementary file 2 — Additional file 2. [file 40662_2024_405_MOESM2_ESM.docx]

**Table S2. A detail description of research in multimodal approaches for age-related macular degeneration.**

| **Author** | **Data preprocessing** | **Data augmentation** | **Model descriptions** | | | | | |  |
| --- | --- | --- | --- | --- | --- | --- | --- | --- | --- |
|  |  |  | **Loss function** | **Learning**  **rate** | **Batch**  **size** | **Epoch** | **Optimizer** | **Model process** | **Public code link** |
| Wang W et al. [45] | - Fundus images were enhanced by contrast-limited adaptive histogram equalization - Median filtering was applied on OCT images for noise reduction | Random rotation, crop, flip, random changes in brightness, saturation and contrast | Cross-entropy | 1e-4 | None | None | SGD with momentum of 0.9 | The net consists  of one branch for  fundus images and one branch for OCT images | None |
| Vaghefi E et al. [46] | - No image processing was performed prior to image analysis | Random crop, horizontalFlip, verticalFlip | Cross-entropy | 1e-4 | None | 100 | Adam | Inception-resnet-v2 design to train multiple image modalities at the same time | <https://medium.com/the-owl/building-inception-resnet-v2-in-keras-from-scratch-a3546c4d93f0> |
| Xu Z et al. [47] | - All images were downloaded in a standard JPEG format - Images were anonymized and preprocessed to remove any artefacts | None | Cross-entropy | None | None | None | SGD with momentum of 0.9, weight decay is 1e-4 | The model extracts a 2048-dim feature vector from each modal in parallel, and are concatenated to form a combined 4096-dim vector for classification | None |
| Chen M et al. [48] | - The paired IR and OCT images were extracted from OCT reports - All images were resized to 280×280 pixels | Random crop, random changing the brightness, contrast, and saturation, random rotation, random horizontally and vertically flip | Cross-entropy | None | None | None | None | Vertical plane feature fusion to fuse features from various modals | None |
| Jin K et al. [49] | - The model resizes OCT and OCTA images to 224×224×3 | None | Cross-entropy | None | None | None | None | Bidirectional fusion network performs two directions of fusion, utilizing the information of two modalities equally | None |
| Chorev M et al. [50] | - Raw images were loaded scan-by-scan by their order in the volume - Images were resized to a unified height of 200 pixels and width of 300 pixels | Affine transformations | Cross-entropy | 3.4e-6 with weight decay of 0.036 | 4 | 100 | Adam | An ensemble model was trained to incorporate all three types of models to achieve a single classifier | None |

OCT = optical coherence tomography; OCTA = optical coherence tomography angiography; IR = infrared reflectance
