# Supplementary material for: Advances and prospects of multi-modal ophthalmic artificial intelligence based on deep learning: a review
Source: Eye Vis (Lond). 2024 Oct 1;11:38. doi: 10.1186/s40662-024-00405-1 (PMC11443922; doi:10.1186/s40662-024-00405-1)
Supplement: Supplementary file 3 — Additional file 3. [file 40662_2024_405_MOESM3_ESM.docx]

**Table S3. A detail description of research in multimodal approaches for diabetic retinopathy.**

| **Author** | **Data preprocessing** | **Data augmentation** | **Model description** | | | | | | |  |  |
| --- | --- | --- | --- | --- | --- | --- | --- | --- | --- | --- | --- |
|  |  |  | **Loss**  **function** | **Learning rate** | **Batch size** | **Epoch** | **Optimizer** | **Model process** | **Public code link** |  |  |
| Li X et al. [58] | None | RandomResizedCrop, RandomGrays-cale, ColorJitter, RandomHorizo-ntalFlip | Cross-entropy | Initial was 1e-4 and dropped by a factor of 0.1 every 1000 epochs | 256 | 2000 | None | - The patient feature-based softmax embedding was a self-supervised signal capturing mutual information across modalities | <https://github.com/xmengli/self_supervised> | | |
| He X et al. [59] | Input images were standardized and normalized | Random horizontally flip | Focal loss | Initial was 1e-3, weight decay is 1e-4 | 8 | 150 | Adam | - Multi-scale attention subnet to extract fundus images’ features - Region-guided attention subnet to extract OCT images’ features - Then cat the features for classification | None | | |
| Li X et al. [60] | None | Random crop, traditional low-level data augmentation | BCE loss | Initial was 1e-2, with the OneCycle adjusted to the learning rate | None | 50 | SGD with momentum of 0.9 and weight decay of 1e-5 | - Instance-level features are fed into the proposed module, which aggregates features into a case-level feature - Then converted into category-wise decision scores | None | | |
| Hervella Á et al. [61] | None | Affine transformation including scaling, rotation, shearing, and color augmentations | Negative structural similarity loss | Constant learning rate of 1e-4 | 1 | 5000 | Adam with decay rates of  *β_1_=*0.9 and *β_2_=*0.999 | - The network designs for the common and exclusive features regarding the input modality and allows the learning rich representations from the unlabeled multimodal visual data | None | | |
| El Habib Daho M et al. [65] | Center cropping on each image was done | Random crop | Cross-entropy | 1e-3 with the OneCycle scheduler | 4 | 200 | AdamW | - Features-level fusion over decision-level fusion was used to capitalize on the rich interplay between the modalities at the feature level | None | | |
| Li Y et al. [66] | None | None | None | None | None | None | None | - Three fusion methods were evaluated: early fusion, intermediate fusion, and hierarchical fusion | None | | |

OCT = optical coherence tomography
